# In This Issue

**Published:** 2003

## THE EPIDEMIOLOGY OF ALCOHOLIC LIVER DISEASE

Alcoholic liver disease (ALD)—and particularly cirrhosis—has long been one of the most prevalent and devastating conditions caused by alcohol consumption, and is one of the leading causes of alcohol-related death. The ALD death rate has declined significantly in recent years, partly as a result of declines in per capita alcohol consumption since the 1970s. According to the authors of this article, Drs. Robert E. Mann, Reginald G. Smart, and Richard Govoni, other factors also appear to be involved in the lower death rates, because mortality rates began to drop when consumption was still increasing. Studies indicate that increased participation in Alcoholics Anonymous and other alcoholism treatment programs has helped reduce cirrhosis mortality. In addition, some research suggests that reduced per capita consumption of spirits, rather than reduced alcohol consumption overall, may account for the fact that cirrhosis rates appeared to decrease even though overall alcohol consumption continued to increase well into the 1970s. Finally, important gender and ethnic-group differences in ALD rates have been found; further research into these differences is likely to lead to improved prevention and treatment of alcohol-related liver disease. (pp. 209–219)

## RELATIONSHIPS BETWEEN NUTRITION, ALCOHOL USE, AND LIVER DISEASE

Excessive alcohol consumption impairs the body’s ability to absorb and use nutrients such as vitamins or proteins and their building blocks, contributing to the malnutrition found in many alcoholics. Dr. Charles S. Lieber reports on the specific effects of excessive alcohol consumption on the digestion of various nutrients, such as proteins and vitamin A, and on the role that these effects can play in the development of alcoholic liver disease. Moreover, the normal metabolism of other nutrients, such as lipids, is impaired by the toxic products generated during alcohol metabolism in the liver, and these toxic products also contribute to liver disease. Finally, Dr. Lieber explores how nutritional approaches, such as various dietary supplements, may assist in the management of alcoholic liver disease. (pp. 220–231)

## HEPATITIS C AND ALCOHOL

For people who are infected with the hepatitis C virus (HCV), heavy drinking heightens the risk of severe liver injury, including cirrhosis and liver cancer. Drs. Eugene R. Schiff and Nuri Ozden review findings from studies of HCV progression and alcohol use and discuss issues in the treatment of alcoholic patients with hepatitis C infection. Although the mechanisms involved in the progression of chronic hepatitis C in alcoholic patients are unclear, evidence suggests that heavy alcohol use may escalate the rate of viral replication and increase viral complexity, hasten liver cell death, promote inflammatory responses, suppress immune responses, and cause accumulation of fat in the liver and of excess iron in body tissues. Drinking heavily during antiviral (e.g., peginterferon) treatment for HCV infection impedes patients’ response to therapy. Abstaining from drinking before and during treatment, however, improves patients’ response to antiviral therapy somewhat (although not totally). Given these findings, the authors recommend that patients be advised to abstain from further alcohol consumption and that patients remain alcohol free for at least 6 months before beginning antiviral therapy. (pp. 232–239)

## HEPATIC ENCEPHALOPATHY

Alcohol-induced liver dysfunction can have consequences for other organs of the body, including the potentially fatal brain disorder hepatic encephalopathy (HE), which is characterized by severe cognitive, psychiatric, and motor disturbances. For the brain to function normally, the liver must remove substances from the blood that can damage brain cells. A liver damaged by excessive alcohol consumption, however, can no longer adequately perform this function, leading to the accumulation of these toxic substances in the blood and, subsequently, the brain. Dr. Roger F. Butterworth summarizes the findings of neuroimaging studies that show excessive ammonia levels as well as manganese deposits in the brains of HE patients; both of these substances may impair brain function in numerous ways and account for the symptoms associated with HE. Several strategies are being evaluated for treating of HE patients. (pp. 240–246)

## DIAGNOSIS AND TREATMENT OF ALCOHOLIC LIVER DISEASE AND ITS COMPLICATIONS

Alcoholic liver disease (ALD) encompasses three conditions: fatty liver, alcoholic hepatitis, and cirrhosis. ALD can be difficult to diagnose. Diagnosis is based on drinking history, physical signs and symptoms, and laboratory tests. However, patients frequently minimize or deny alcohol abuse, there may be no evidence of ALD from the physical exam, and laboratory abnormalities may not specifically point to ALD. In this article, Drs. Luis S. Marsano, Christian Mendez, Daniell Hill, Shirish Barve, and Craig J. McClain explain issues relevant to the diagnosis of ALD and explore the treatment of ALD and its complications. Current treatment strategies include lifestyle changes to reduce alcohol consumption, cigarette smoking, and obesity; nutrition therapy; and pharmacological therapy. As the authors explain, diagnosing and managing the complications of ALD also are important for decreasing the duration of sickness, improving quality of life, and decreasing mortality. (pp. 247–256)

## LIVER TRANSPLANTATION FOR ALCOHOLIC LIVER DISEASE

Liver transplantation is the only definitive treatment currently available for patients with end-stage liver disease, but liver transplantation for patients with alcoholic liver disease remains controversial, mainly because of concern that alcoholics may resume drinking after the procedure, thereby potentially “wasting” valuable donor organs. According to Drs. Abhinandana Anantharaju and David H. Van Thiel, however, a comprehensive evaluation of potential transplant candidates can help identify suitable patients who are likely to remain abstinent and comply with the medical regimen after the procedure. Particularly when such a screening process is implemented, the outcome of patients with alcoholic liver disease in terms of survival, rejection of the transplanted organ, sustained sobriety, and other variables appears to be comparable to that of patients with non-alcoholic liver disease. (pp. 257–269)

